# Development and Initial Validation of High-Fidelity 3D Hybrid Simulators for Training in Complex Pediatric Oncologic Surgery

**DOI:** 10.3390/cancers18193135

**Published:** 2026-09-28

**Authors:** Pamela Lustig, Arnau Valls-Esteve, Carlos Alaez, Albert Pasten, Lucas Krauel

**Affiliations:** 1 3D4H Unit, SJD Barcelona Children’s Hospital, Universitat de Barcelona, 08950 Esplugues de Llobregat, Spain; pamela.lustig@sjd.es (P.L.); arnau.valls@sjd.es (A.V.-E.); 2 Innovació en Tecnologies Sanitaries, Institut de Recerca Sant Joan de Déu (IRSJD), Santa Rosa 39-57, 08950 Esplugues de Llobregat, Spain; 3 Simulation Unit, SJD Barcelona Children’s Hospital, Universitat de Barcelona, 08950 Esplugues de Llobregat, Spain; carlos.alaez@sjd.es; 4 Pediatric Surgery Department, SJD Barcelona Children’s Hospital, Universitat de Barcelona, 08950 Esplugues de Llobregat, Spain; apastego44@alumnes.ub.edu; 5 Sarcomes i Neuroblastoma, Institut de Recerca Sant Joan de Déu (IRSJD), Santa Rosa 39-57, 08950 Esplugues de Llobregat, Spain

**Keywords:** pediatric oncology, surgical simulation, 3D printing, nephron-sparing surgery, Wilms tumor, neuroblastoma, image-defined risk factors, team-based surgical training, platinum-cure silicone

## Abstract

**Background:** This study reports the development and initial face and content validation of high-fidelity 3D hybrid simulators for training in complex pediatric oncologic surgery. Pediatric solid tumor surgery is technically demanding; case volumes are low, and procedural errors carry lifelong oncologic or functional consequences. High-fidelity simulation offers a route to structured training without patient risk. **Methods:** Patient-derived hybrid simulators were developed by combining 3D-printed anatomical molds with multi-hardness platinum silicone casting. Three oncologic scenarios were reproduced: nephron-sparing surgery (NSS) for Wilms tumor, neuroblastoma L1, and neuroblastoma L2 with major vascular involvement. Retrospective imaging datasets were segmented using Philips IntelliSpace Portal to generate patient-derived STL files. Bony components (simulator box, thoracic wall) were fabricated by selective laser sintering (SLS) in PA12 polyamide. Silicone casting molds were produced by fused filament fabrication (FFF) in basic PLA. Silicone selection (Smooth-On Ecoflex and Dragon Skin series) was based on our team’s expertise in the mechanical characteristics of human organs, matching manufacturer-provided Shore hardness datasheet values to the closest available reference for each tissue type. All components were assembled in anatomically accurate sequence within a patient-sized housing box. Validation was conducted through structured workshops with fifteen pediatric surgical oncologists using a 5-point Likert scale. **Results:** Mean Likert scores, calculated from participant-level composite domain scores (*n* = 27 model evaluations from 15 participants), were high across all domains: anatomical accuracy 4.14 ± 0.53 (median 4.00, IQR 3.67–4.67), dissection realism 4.29 ± 0.45 (median 4.33, IQR 4.00–4.50), and tactile fidelity 4.37 ± 0.56 (median 4.00, IQR 4.00–5.00). The L2 neuroblastoma model was rated particularly valuable for rehearsing synchronized two-surgeon workflows and anticipatory communication during high-risk vascular dissection. **Conclusions:** These hybrid simulators provide a reproducible, high-fidelity platform with strong expert-perceived realism, supporting rehearsal of technical maneuvers and coordinated two-surgeon workflows; formal evaluation of skill acquisition and clinical transfer remains warranted.

## Simple Summary

Surgery for children with certain rare cancers like Wilms tumors and neuroblastoma can be extremely difficult to operate on because these tumors often grow close to major blood vessels or vital organs. Because these cancers are uncommon, surgeons have few opportunities to practice the specific techniques needed before operating on a real child. To help address this gap, we created realistic, hands-on training models reproducing three particularly challenging types of these operations. Each model was built using 3D printing and specially designed silicone materials chosen to look, feel, and behave like real organs and tumors, based on imaging from real children. Fifteen pediatric cancer surgeons tested the models by performing simulated operations on them. They rated the models highly for how realistic the anatomy, tissue, and surgical experience felt and found them especially useful for practicing how two surgeons can work safely together during the most complex procedures. These findings suggest that such models offer a safe, repeatable way for surgical teams to prepare rare, high-risk operations before performing them on children, with the goal of making these surgeries safer and more successful for young patients.

## 1. Introduction

Pediatric solid tumors arise within the context of ongoing organ development and display distinct biological behavior compared to adult malignancies. Over the past decades, multimodal strategies integrating surgery, chemotherapy, radiotherapy, and molecular risk stratification have substantially improved survival. Cooperative group trials have increased overall survival in favorable-histology Wilms tumor to above 90%, establishing pediatric oncology as a paradigm of protocol-driven, multidisciplinary success [1,2].

Despite these advances, surgery remains a critical determinant of oncologic outcomes. In several pediatric solid tumors, the quality, safety, and completeness of resection directly influence survival and long-term morbidity. As systemic therapies continue to evolve, surgical objectives increasingly extend beyond tumor eradication toward functional preservation and minimization of late sequelae.

### 1.1. Wilms Tumor and the Precision of Nephron-Sparing Surgery

Wilms tumor (nephroblastoma) accounts for more than 90% of renal malignancies in children, with an overall incidence of approximately one per million children [1]. Treatment approaches differ between cooperative groups: while the Children’s Oncology Group (COG) traditionally favors upfront nephrectomy followed by adjuvant chemotherapy, the UMBRELLA SIOP-RTSG 2016 protocol recommends preoperative chemotherapy for most patients to reduce intraoperative tumor rupture risk and facilitate resection [3]. Despite these differences, both groups converge on a chemotherapy-first strategy when bilateral disease is present—identified in approximately 5% of cases at diagnosis—followed by nephron-sparing surgery (NSS), with the goal of maximizing oncologic control while preserving residual renal function [1,4]. Standard treatment in unilateral cases comprises nephrectomy with accurate lymph node sampling; however, the increasing recognition of bilateral disease, genetic predisposition syndromes, and long-term nephron-cardiovascular risk has expanded the role and importance of NSS in selected patients.

NSS aims to achieve oncologically complete tumor removal while preserving maximal functional renal parenchyma. Unlike total nephrectomy, it requires detailed three-dimensional understanding of intrarenal tumor boundaries, segmental vascular anatomy, and the collecting system. Tumor spill may upstage disease and intensify adjuvant therapy, whereas excessive parenchymal sacrifice may predispose to chronic kidney disease and cardiovascular morbidity [4,5]. Intraoperative decisions are therefore oncologically consequential, and the technical margin for error is narrow. Centralization of care and the use of preoperative 3D modeling have been associated with improved outcomes [5].

### 1.2. Neuroblastoma and Image-Defined Risk Factors

Neuroblastoma (NB) is the most common extracranial solid tumor of childhood, arising from the sympathetic nervous system with an estimated incidence of one per 100,000 infants [6,7]. Approximately 75% of primaries develop in the abdomen. Tumor staging is based on image-defined risk factors (IDRFs) defined by the International Neuroblastoma Risk Group (INRG), which reflect encasement of major vessels and involvement of vital structures [7].

In IDRF-positive (L2) disease, resection often necessitates circumferential dissection around the aorta, inferior vena cava, celiac axis, or superior mesenteric vessels combined with extensive organ mobilization. Although multimodal therapy is standard in high-risk cases, gross-total resection is recommended as soon as the tumor appears resectable on imaging [6]. Three-dimensional preoperative planning has been shown to improve understanding of tumor-vessel relationships in anatomically complex cases [8,9]. These operations demand meticulous vascular awareness and continuous adjustment of traction, counter-traction, and exposure, rendering coordinated two-surgeon workflows critical for operative safety.

### 1.3. The Educational Gap in High-Risk Pediatric Oncologic Surgery

Pediatric solid tumors are rare. Even in tertiary referral centers, the number of complex NSS or IDRF-positive neuroblastoma resections available for training is limited. Workforce data underscore the limited case volumes in pediatric surgical subspecialties [10], and traditional apprenticeship models are insufficient for procedures in which intraoperative errors may compromise oncologic control or produce lifelong morbidity. Structured surgical safety frameworks specifically addressing error traps in neuroblastoma, hepatoblastoma, and Wilms tumor resection underscore the need for deliberate training beyond apprenticeship [11].

Surgical simulation shortens learning curves and enhances patient safety [12]. Human cadavers offer anatomical fidelity but are scarce and cannot replicate tumor-related distortion; animal models cannot reproduce tumor-specific pathology, and their divergent anatomy together with associated bioethical concerns further limits their applicability to oncologic scenarios such as Wilms tumor or neuroblastoma; and standard 3D-printed rigid models lack biomechanical realism for tissue dissection [12]. Options exist for training in specific pediatric minimally invasive techniques—pyloromyotomy [13], choledochal cyst excision [14], inguinal hernia repair [15], and open neonatal procedures [16,17] —but high-fidelity simulators for open abdominal oncologic resections in children remain scarce.

In the field of oncology, simulators for NSS have been described for robotic-assisted procedures in adults [18] and, recently, for open Wilms tumor resection using hydrogel phantoms [4]. Our group has previously reported technical workflows for multi-material model fabrication in pediatric oncology [9,19] and the use of 3D prototypes for preoperative planning in tumors with vascular encasement [8]. However, no consensus exists on the most realistic materials for simulating renal parenchyma and tumor tissue, and existing simulators appear to focus primarily on specific surgical techniques or approaches rather than on the comprehensive training of complex open neuroblastoma or NSS resections.

Complex pediatric oncologic surgery is not merely a technical act but a coordinated cognitive process requiring shared operative planning, anticipatory vascular risk management, and synchronized interaction between surgeons. Building on prior work demonstrating the value of patient-derived 3D models for surgical planning in cases with vascular encasement [8] and on established workflows for soft silicone model fabrication [20], we developed hybrid simulators integrating 3D-printed anatomical scaffolds with multi-hardness silicone casting to recreate three high-complexity oncologic scenarios: NSS for Wilms tumor, neuroblastoma L1, and neuroblastoma L2 with major vascular involvement. We aimed to evaluate not only anatomical and tactile realism but also the capacity of these simulators to facilitate structured training in coordinated two-surgeon workflows.

## 2. Materials and Methods

### 2.1. Study Design and Simulator Concept

Hybrid surgical simulators were developed and validated at Hospital Sant Joan de Déu (Barcelona, Spain), a tertiary pediatric referral and university hospital. The simulators were designed to replicate three high-complexity oncologic scenarios: (1) NSS for Wilms tumor; (2) L1 neuroblastoma resection (localized, without IDRF involvement of major vessels); and (3) L2 neuroblastoma resection with circumferential encasement of major abdominal vessels. For greater practicality and to facilitate structured training, the simulator was divided into left and right anatomical sides. The neuroblastoma model was placed on the patient’s left side, while the Wilms tumor model was positioned on the right side. This spatial organization was intentionally designed to standardize the training setup and enable a more comprehensive and comparative learning experience across oncologic scenarios. Development was conducted in close collaboration between the 3D Printing for Health Unit (3D4H), the pediatric surgical oncology team, and the advanced simulation program of the institution.

Each simulator integrates patient-derived anatomical molds produced by additive manufacturing with multi-durometer silicone casting to replicate the biomechanical properties of abdominal soft tissues. The overarching goal was to achieve anatomical fidelity derived from real patient imaging and tactile fidelity grounded in published tissue hardness data, enabling both individual technical skill rehearsal and structured training in coordinated two-surgeon operative workflows.

To enhance realism and surgical complexity, the Wilms tumor model was designed to contain two distinct tumor foci: a superficial lesion, minimally visible on the renal surface, and a deeper intraparenchymal lesion detectable only by intraoperative ultrasound and located in direct contact with the renal calyces. Each focus was cast at a different Shore hardness, reflecting the heterogeneous consistency encountered in actual Wilms tumors, so that surgeons could perceive distinct tactile feedback during palpation and dissection of each lesion. This configuration was intended to reproduce the diagnostic and technical challenges of nephron-sparing surgery, requiring surgeons to integrate ultrasonographic localization with careful dissection to achieve complete resection while preserving the collecting system and maximal functional parenchyma (Figure 1).

### 2.2. Case Selection and Retrospective Imaging Analysis

A retrospective review of pediatric oncology cases treated at the institution was performed to characterize the range of tumor morphologies, vascular relationships, and anatomical distortions encountered in clinical practice. For neuroblastoma, cases were classified according to the INRG staging system with particular attention to IDRFs. For Wilms tumor, cases with indications for NSS were reviewed, including bilateral disease, solitary kidney, and genetic predisposition syndromes.

Three representative scenarios were selected to anchor simulator design. The neuroblastoma simulators were calibrated to approximate the anatomy of a two-year-old patient, consistent with the peak incidence age for abdominal neuroblastoma (12 months to 4 years). Anatomical structures, including kidneys, vasculature, liver, backbone, retroperitoneum, and spleen, were derived from patient imaging datasets. The tumor mass for each scenario was derived from real patient imaging; the workflow is fully capable of exact, unmodified patient-specific replication when clinically indicated (e.g., for individual preoperative rehearsal). For the present validation study, the source imaging was deliberately and modestly modified for didactic purposes to optimize educational representativeness: in the Wilms tumor model, two distinct tumor foci of differing depth and Shore hardness were introduced to represent a range of surgical difficulty within a single scenario; in the L1 neuroblastoma model, tumor size was slightly increased relative to the source case; and in the L2 neuroblastoma model, the anatomy was modified to incorporate involvement of the renal vessels, celiac trunk, and aorta, reproducing a representative high-complexity IDRF-positive scenario. This approach allowed deliberate design of surgically challenging tumor-vessel relationships specific to each educational objective while preserving the patient-specific origin of the underlying anatomy.

### 2.3. Medical Image Segmentation and 3D Reconstruction

Medical imaging datasets in DICOM format were processed using Philips IntelliSpace Portal (version 12; Philips Healthcare, Amsterdam, The Netherlands), the clinical imaging platform of the institution. Contrast-enhanced computed tomography (AngioTC) was the preferred modality for neuroblastoma cases, owing to its superior visualization of vascular displacement and collateral vessel formation caused by tumor encasement.

Segmentation was performed using a semi-automatic region-growing algorithm, which groups voxels based on intensity similarity and halts at boundaries where the similarity criterion is no longer met. Given the inherently similar Hounsfield densities of abdominal soft tissues, segmentation of parenchymal organs, retroperitoneal structures, and tumor masses required expert manual refinement with support from a dedicated pediatric oncology radiologist. Structures segmented included bilateral kidneys, liver, spleen, abdominal aorta with its major branches (celiac axis, superior mesenteric artery, and renal arteries), inferior vena cava, and the thoracic and pelvic bony skeleton.

The tumor mass for each scenario was sculpted digitally in Meshmixer (Autodesk, San Francisco, CA, USA) to reflect the morphological and vascular relationships characteristic of the corresponding surgical scenario. In the L1 model, the tumor was positioned lateral to the great vessels without encasement. In the L2 model, the tumor was designed to wrap the anterior surface of the aorta and encase the origin of the ipsilateral renal artery, consistent with IDRF criteria. (Figure 2a) In the Wilms tumor NSS model, two distinct tumor foci were designed to increase realism and surgical complexity: a superficial lesion, minimally visible at the renal surface, and a deeper intraparenchymal lesion located at the superior renal pole with hilar proximity and in direct contact with the renal calyces, detectable only by intraoperative ultrasound (Figure 2b). This configuration was intended to challenge surgeons rehearsing ultrasonographic localization and parenchymal preservation. All segmented and synthesized structures were exported as STL files for import into the CAD environment.

### 2.4. Simulator CAD Design and Mold Engineering

Three-dimensional modeling was conducted in Rhinoceros 3D (version 8; Robert McNeel and Associates, Seattle, WA, USA), a NURBS-based CAD environment capable of importing and editing STL mesh files alongside parametric surface geometry. The imported anatomical meshes were spatially positioned within a custom-designed simulator housing calibrated to the torso proportions of a two-year-old patient (footprint 216.9 mm × 255.0 mm; depth 138.7 mm including lid). The housing incorporated the bony thoracic wall and pelvic brim as integral rigid elements, reflecting anatomical boundaries that define the operative field and provide retraction landmarks for surgical trainees.

Silicone casting molds were designed using a negative-cavity strategy for all parenchymal organs. Each mold incorporated an 8 mm diameter inlet channel for silicone injection via syringe and an air outlet channel positioned at the highest anatomical point of the cavity, preventing air entrapment and bubble formation during casting. A precision-fit alignment lip was incorporated into all mold halves, ensuring single-orientation assembly and preventing displacement during curing (Figure 3). CAD volume measurements were used to calculate the exact silicone volume required for each component, with a 20% surplus to ensure complete fill, including channels. Blood vessels required a positive lumen mold strategy: a water-soluble PVA mold was printed to create a hollow lumen within the silicone wall, subsequently dissolved in water to yield patent tubular structures (Section 2.6).

### 2.5. Material Selection: Tissue Biomechanics and Shore Hardness Matching

Material selection was guided by a review of published tissue mechanical data. All abdominal soft tissues fall within the Shore 000 to Shore 00 hardness range, distinct from the harder Shore A range of denser biological materials. Target hardness values for each anatomical structure were extracted from the literature, with reference to Tejo Otero et al. [19], who characterized Shore hardness of abdominal organs, and Maclean et al., [21] who reported Shore 00 values for aortic tissue. Adams et al. [22] provided comparative elastic modulus data confirming that platinum-cure silicone elastomers (approximately 60 kPa) match native renal parenchyma far more closely than agarose, PVA hydrogels, or commercially available flexible 3D-printable polymers such as TangoPlus (Stratasys), whose elastic modulus is approximately 20-fold higher than kidney tissue.

Platinum-cure silicones from the Smooth-On product line (Smooth-On Inc., Macungie, PA, USA) were selected for all soft-tissue components based on their wide Shore hardness range (Shore 00-10 through Shore A 30), translucency allowing tissue-realistic pigmentation with SilTone pigments (Smooth-On), chemical stability without time-dependent property loss (as occurs with hydrogels), and compatibility with surgical instruments. In the case of the blood vessels, fast-curing silicone was used to ensure that the silicone coating set rapidly, preventing any deformation during curing due to the constraints of the internal PVA mold.

Because commercially available silicone formulations do not always provide an exact match to the hardness values reported for biological tissues, material selection was based on the combination of published reference data, available commercial formulations, and iterative tactile assessment by the internal surgical team. The literature values were therefore considered target reference ranges rather than exact material specifications. When an exact match was not commercially available, the closest suitable formulation was selected and subsequently evaluated by the surgical team based on its perceived tactile similarity to the corresponding anatomical tissue.

Table 1 summarizes Shore hardness targets and corresponding silicone selections for each anatomical structure.

Silicone pigmentation was performed using SilTone pigments, which are specifically formulated for platinum-cure systems and do not inhibit the curing reaction. Color selection was guided by the surgical team’s direct clinical experience and intraoperative photographic documentation reviewed during iterative development workshops. The neuroblastoma tumor was rendered with a yellowish base tone with marbled vascular patterns, achieved by incorporating loose wool fiber fragments into the uncured silicone before casting to simulate the macroscopic appearance of tumor vascularity.

### 2.6. Additive Manufacturing: FFF Mold Fabrication and SLS Box Production

Two complementary additive manufacturing technologies were employed, selected on the basis of geometric requirements and functional roles.

Fused Filament Fabrication (FFF). All silicone casting molds were produced by FFF using a Bambu Lab X1 Carbon printer (Bambu Lab, Shenzhen, China) with a 0.4 mm nozzle. Uniform printing parameters were applied across all mold components: layer height 0.16 mm, infill density 15%, and printing temperature 200 °C. Molds for parenchymal organs (kidneys, liver, spleen, tumor, urinary tract, and intervertebral discs) were printed in PLA (Bambu Lab PLA Basic; elongation at break 12.2%), providing dimensional stability, low warping, and a clean surface finish. Water-soluble PVA filament (Bambu Lab, Shenzhen, China) was used exclusively for the positive lumen-forming mold of the vascular structures, exploiting its water solubility to achieve hollow tubular geometries without mechanical mold extraction. STL files were sliced using Bambu Studio software (Version 2.4.0.70) (Bambu Lab, Shenzhen, China). Total accumulated FFF print time across all molds was approximately 47 h 31 min, with PLA consumption of approximately 1.3 kg and 80 g of PVA.

Selective Laser Sintering (SLS). The simulator box, integrated thoracic wall, and lid were fabricated by SLS using a RICOH AM S5500P industrial system (RICOH Company, Ltd., Tokyo, Japan; build volume 550 × 550 × 500 mm) with PA12 polyamide powder. SLS was selected for these components because the complex curved geometry of the thoracic cage—with undercut rib contours and non-planar surfaces—required support-free fabrication intrinsic to powder-bed technology. PA12 provides mechanical rigidity for repeated simulator handling, dimensional accuracy for bony landmark reproduction, and a matte surface consistent with cortical bone appearance. SLS fabrication was performed at an accredited external facility (CIM UPC, Barcelona, Spain) (Figure 4).

### 2.7. Silicone Casting Protocol

All platinum-cure silicones were processed according to a standardized casting protocol. Each formulation is a two-component system (Part A and Part B) mixed at a 1:1 ratio by weight. The appropriate tissue-matching pigment was blended homogeneously into Part A before the addition of Part B. For structures requiring a marbled vascular appearance (tumor, viscera), a drop of contrasting pigment was introduced after combining both components and blended minimally to generate the marbled effect. A mold-release agent (Ease Release 200, Smooth-On) was applied to all PLA mold surfaces prior to casting to prevent adhesion.

The silicone mixture was loaded into a 100 mL syringe and injected through the mold inlet channel until material emerged from the air outlet, confirming complete cavity fill. Molds were clamped and allowed to cure at room temperature (23 °C) for the manufacturer-specified time, then kept closed overnight before demolding.

Vascular structures required a dedicated hollow-tube casting procedure. The PVA lumen mold was coated with Dragon Skin 10 Fast (pot life 8 min) by progressive rotation during pouring, building a uniform silicone wall. Once the silicone reached a clay-like consistency at the end of the pot life, the outer surface was manually refined to simulate adventitial texture. The number of coating layers applied differed between the vena cava and the aorta, with the aorta being slightly thicker. After full cure, the PVA scaffold was immersed in warm water for 48 h with regular water exchange until complete dissolution, yielding patent, compliant hollow silicone vessels (Figure 5).

In the case of Wilms tumor, several molds were designed to cast the different internal anatomical structures of the kidney (ureter, collecting system, and tumor). In an initial stage, both Wilms tumors and the collecting system were cast separately using the selected silicone. Once these components were formed, they were placed inside the kidney mold, and silicone matching the hardness of the renal tissue was poured to complete the structure. Additionally, to make the tumors detectable with an ultrasound scanner, the silicone used for the Wilms tumors was loaded with corn flour [23]; this increased its density and ensured reliable visualization under ultrasound. (Figure 6).

### 2.8. Simulator Assembly

Assembly proceeded sequentially from the SLS-printed box as a structural base. Intervertebral disc components were placed between the printed vertebral bodies. Soma Foama 25 tinted with red pigment was then cast directly into the box to form the dorsal muscle layer, which simultaneously served as a compliant support bed for organ placement and replicated the soft resistance encountered during retroperitoneal exposure.

The vascular core assembly was constructed by positioning and bonding both kidneys, the vascular tree (aorta, inferior vena cava, and renal vessels, mesenteric and celiac trunk), and the tumor mass, using SilPoxy silicone adhesive (Smooth-On) for all component-to-component and component-to-box fixation. Tumor positioning was verified against the original CAD assembly for each scenario. Vascular structures and ureters were maintained under slight tension by fixation at the cranial and caudal ends of the box, reproducing the taut tissue resistance encountered when applying retraction during vessel dissection.

The liver and spleen were positioned in anatomical location following vascular core assembly, replicating their role as structures requiring mobilization to access the retroperitoneal operative field—specifically the Kocher maneuver for right-sided exposure and hepatic retraction. The completed intestinal package was placed as the most superficial layer. The simulator was closed with the SLS-printed lid for transport and storage. Table 2 summarizes the manufacturing approach for each anatomical component.

### 2.9. Validation Protocol

Validation was conducted through structured workshops at the pediatric surgical oncology center. Fifteen board-certified pediatric surgical oncologists, all with clinical dedication to pediatric surgical oncology at their respective centers, participated; none had prior exposure to the developed simulators or to any oncologic surgical simulation platform. Consistent with the rarity of these procedures and the absence of prior simulators of this kind, this reflects the field rather than a selection bias; all participants were actively involved in surgical education and training at their institutions. Not all participants evaluated every model: the Wilms tumor NSS and neuroblastoma L1 models were each assessed by a subset of the cohort (*n* = 10 and *n* = 12, respectively), while the neuroblastoma L2 scenario, reproducing circumferential vascular encasement, was evaluated across two workshop sessions with different senior-surgeon operating configurations (*n* = 5 individual evaluator responses in total), consistent with its design as a higher-complexity, coordinated assessment. Both sessions preserved the intended two-surgeon operative workflow at any given time: in one session, three senior surgeons rotated through the two active operating roles during the procedure, while in the other, two surgeons performed the full resection together. The three Likert domains were scored separately for each model by the participants who evaluated it; one of the authors (L.K.) participated as an operating partner in the paired L2 sessions only—he had no operative or evaluative role in the Wilms or L1 models—and completed the evaluation questionnaire for one of those L2 sessions, representing a source of assessment bias given his role in developing the model. Because working alongside a developer-author may also have influenced the other, external evaluators’ ratings, we performed a sensitivity analysis excluding this participant’s response: L2 domain scores changed minimally (anatomical accuracy 3.87 → 3.92; dissection realism 4.09 → 4.06; tactile fidelity 4.20 → 4.25 for *n* = 4 vs. *n* = 5), indicating that the reported L2 findings are not materially dependent on this participation. Reflecting the scheduling constraints of a single-day workshop, participant-level coverage overlapped substantially across models rather than reflecting three independent, non-overlapping panels: one participant evaluated all three models, most participants evaluated both the Wilms tumor NSS and neuroblastoma L1 models, and the neuroblastoma L2 scenario was evaluated by a partially overlapping subset of senior surgeons assigned to paired sessions. The validation questionnaire also included an open-ended feedback section consisting of three questions addressing the main benefits of the neuroblastoma simulator for training, the challenges or limitations encountered during its use, and suggestions for future improvements. Overall, participants highlighted the realism of the tissues, anatomical relationships, and three-dimensional visualization as the main strengths of the simulator, particularly for practicing tissue handling, dissection, and surgical decision-making. The main limitation identified was the realism and handling of vascular structures. Suggested improvements included enhancing vessel anatomy and consistency, introducing simulated bleeding or fluid within vessels, increasing case difficulty, and incorporating cases with vascular encasement or vessels suitable for diathermy use. Participants performed complete or partial simulated resections on each model using standard open surgical instrument sets.

Realism and educational value were assessed using a five-point Likert scale (1 = poor, 5 = excellent) across three primary domains: anatomical accuracy (spatial relationships of tumor to adjacent organs and vessels), dissection realism (tissue resistance, plane development, and tissue behavior under instrument forces), and tactile fidelity (haptic properties of individual tissue components). Anatomical accuracy and dissection realism were each assessed using several related questionnaire items (composited for analysis as the participant-level mean), while tactile fidelity was assessed with a single item. The complete questionnaire, comprising seven sections (general background, anatomical accuracy, realism of procedural steps, simulator experience, educational value, comparison with other training methods, and open-ended feedback), is provided as Supplementary Material S1. The L2 neuroblastoma scenario was additionally validated across two workshop sessions with senior surgeons to assess its capacity to facilitate coordinated two-surgeon workflows, with specific focus on shared spatial awareness, synchronized retraction and dissection, and anticipatory communication (Figure 7). Qualitative feedback was collected in structured debriefs following each simulation session. Quantitative data are presented as means and standard deviations. Imaging data used for simulator design and fabrication were completely anonymized and obtained from the hospital’s institutional imaging repository, for which patients (or legal guardians) had provided informed consent covering the use of imaging data for teaching and/or scientific purposes. As the subjects of the present validation study were the participating surgeons rather than the patients whose anonymized imaging informed model design, no additional institutional ethics committee review was required for this study.

### 2.10. Iterative Development Process

Simulator development followed an iterative design-fabricate-evaluate cycle. Each prototype iteration was assessed by the core surgical team for anatomical fidelity, tactile properties, and operative realism. Feedback was systematically incorporated into subsequent revisions, including adjustments to silicone formulation, pigmentation, organ positioning, tumor morphology, and vascular tension. This process ensured that final simulator versions reflected clinical consensus on the surgical scenarios they were designed to replicate.

## 3. Results

Overall, participants reported high levels of anatomical realism and educational value. The models were particularly valued for enabling rehearsal of coordinated dissection strategies, exposure techniques, and vascular risk management in anatomically complex scenarios.

Quantitative evaluation using a five-point Likert scale demonstrated high mean scores across all assessed domains (Table 3):

Likert scores were additionally disaggregated by simulator model rather than reported only as a pooled mean across the three scenarios (Table 4). Domain-level scores were highest for the Wilms tumor NSS and neuroblastoma L1 models, with modestly lower scores for the neuroblastoma L2 model, particularly for dissection realism, consistent with its greater anatomical and technical complexity.

Because most participants evaluated more than one simulator model within the same workshop session, the domain scores summarized by model in Table 4 are not fully independent across models; Table 5 summarizes participant-level coverage across the three scenarios (*n* = 15 unique participants). For the neuroblastoma L1 model, two participants who worked as a pair submitted a single joint response, which is counted as one of the twelve response units summarized in Table 4 for that model; both participants are counted individually in Table 5.

In the neuroblastoma L2 scenario—validated by pairs of senior surgeons—participants emphasized the model’s capacity to reproduce vascular relationships and the requirement for coordinated organ mobilization. This scenario was perceived as particularly valuable for rehearsing synchronized two-surgeon workflows and anticipatory communication during high-risk vascular dissection.

Qualitative feedback identified the physical reproduction of the retroperitoneal anatomy, the resistance and behavior of the silicone organ surrogates under surgical instrument forces, and the spatial authenticity of tumor-vessel encasement as the principal drivers of perceived realism. Several participants noted that the L2 neuroblastoma scenario offered training in coordinated exposure and dissection sequences that are difficult to rehearse in any existing training platform (Figure 8).

## 4. Discussion

The principal finding of this study is that patient-derived hybrid simulators integrating 3D-printed molds and multi-durometer silicone can faithfully reproduce the anatomical relationships, tissue biomechanics, and operative complexity of NSS for Wilms tumor and neuroblastoma resections across both L1 and L2 risk categories. High scores for anatomical accuracy, dissection realism, and tactile fidelity corroborate this across three independent surgical scenarios.

### 4.1. Team-Based Surgical Cognition

Beyond individual technical skill acquisition, one of the most significant contributions of these simulators was the ability to rehearse coordinated two-surgeon workflows under conditions that closely replicate real oncologic surgery. Complex pediatric tumor resections are not isolated technical acts: exposure, countertraction, vascular control, and tumor dissection occur simultaneously and require continuous micro-adjustments between operators. The operative field is shared, anatomical planes are distorted, and minor misalignment in force application or instrument positioning may increase the risk of tumor spill or vascular injury.

In the NSS model, surgeons reported that rehearsal refined strategies for coordinated parenchymal incision, vascular identification, and margin control while preserving renal tissue. The spatial relationship between tumor mass, segmental vessels, and collecting system encouraged anticipatory positioning and synchronized instrument handling—interactions that are difficult to conceptualize in static models or cadaveric training and are seldom explicitly taught.

The impact was even more evident in the L2 neuroblastoma scenario, where safe resection demands mobilization of adjacent organs and stable exposure of circumferentially involved vascular structures. Qualitative feedback from participants suggested that the simulator could enable surgeons to practice what may be described as operative choreography, a hypothesis-generating concept extending beyond manual dexterity to encompass the shared mental model of the operative field that allows anticipatory assistance rather than reactive correction. When one surgeon advances dissection, the other adjusts exposure in anticipation of the next maneuver. The simulator environment allowed this anticipatory behavior to emerge in a structured and reproducible manner. This remains a qualitative, hypothesis-generating observation, as no standardized coordination or communication metric was applied.

### 4.2. Cognitive Load Distribution

A single surgeon managing exposure, dissection, and vascular monitoring may experience cognitive overload, increasing the probability of error in high-risk resections. Coordinated dual-operator workflows distribute perceptual and decision-making demands, potentially enhancing procedural safety. By replicating realistic anatomical resistance and vascular relationships, the simulators provided a platform in which this cognitive distribution could be rehearsed without patient risk. These qualitative observations, based on structured debriefs rather than a standardized teamwork assessment instrument, suggest the hypothesis that high-fidelity oncologic simulation may serve not only as a tool for individual technical training but also as an environment for rehearsing team-based surgical cognition; this hypothesis warrants formal evaluation with objective coordination or communication metrics.

### 4.3. Comparison with Existing Simulation Platforms

Existing pediatric simulation platforms address minimally invasive techniques [13,14] and select open procedures such as neonatal laparotomy [16] and open pyeloplasty [17]. In oncologic surgery, hydrogel phantoms have been described for NSS in Wilms tumor [4], and perfused robotic partial nephrectomy platforms exist for adult patients [18]. These approaches offer valuable advantages, including realistic tissue handling and, in some cases, the ability to reproduce specific surgical techniques or perfusion characteristics. Hydrogels offer excellent short-term tissue fidelity but exhibit progressive property loss due to dehydration, limiting reusability and reproducibility across training sessions. The platinum-cure silicone approach adopted here offers comparable tactile properties with indefinite shelf stability.

Our group has previously reported multi-material fabrication workflows for complex pediatric oncologic anatomical models [9,19] and the use of 3D prototypes for preoperative planning in vascular encasement cases [8]. The present work extends this line of research by specifically targeting procedural simulation and validated team-based training, moving beyond preoperative planning tools toward a structured competency-based educational platform. To the best of our knowledge, these are the first high-fidelity simulators validated for open pediatric oncologic resections encompassing both a renal and two neuroblastoma scenarios and the first to explicitly address coordinated two-surgeon workflow development.

### 4.4. Limitations and Future Directions

The current models do not incorporate dynamic perfusion, limiting simulation of intraoperative bleeding and hemorrhage control—a critical skill in high-risk vascular dissection. Future iterations will integrate perfusion circuits to add physiological realism in this domain.

Formal assessment of objective structured assessment of technical skills (OSATS) and transfer of training to clinical performance were not evaluated in this initial face-validity study and represent important objectives for subsequent research. The number of participants, while representative of the targeted expert subspecialty, remains limited; multicenter validation with a larger cohort is warranted to establish construct validity and generalizability. No formal a priori sample size calculation was performed; the number of participants (*n* = 15) reflects the availability of pediatric surgical oncologists with the relevant subspecialty expertise at our institution and collaborating centers, consistent with similar early-stage simulator validation studies. Shore hardness matching for each anatomical structure was based on manufacturer-specified silicone datasheets and published tissue biomechanical reference data rather than independent mechanical testing (e.g., durometer verification or tensile/compression testing) of the cast materials; independent mechanical validation is planned for future iterations of the platform. A formal stratified analysis of realism scores by participant experience level was not performed given the sample size and represents a direction for future multicenter validation with larger cohorts. Finally, because most participants rated more than one simulator model (Table 5), the domain scores reported by model in Table 4 are not fully independent observations; this overlapping design was a practical consequence of workshop scheduling rather than a randomized or counterbalanced allocation and should be considered when interpreting between-model comparisons.

## 5. Conclusions

We developed and initially validated high-fidelity hybrid simulators replicating NSS for Wilms tumor and neuroblastoma resections (L1 and L2), integrating patient-derived 3D-printed anatomical molds with multi-durometer platinum-cure silicone casting to reproduce realistic anatomical relationships and tissue biomechanics, and obtained expert-based validation from pediatric surgical oncologists.

These models provide a reproducible platform for rehearsal of critical technical maneuvers, precise parenchymal preservation in NSS, and vascular dissection in IDRF-positive neuroblastoma and appear promising for structured training in coordinated two-surgeon operative workflows, a hypothesis that warrants formal evaluation. In rare and technically demanding pediatric oncologic procedures where clinical exposure is inherently limited, high-fidelity team-based simulation may represent a valuable strategy to help address the learning curve without exposing patients to risk, pending formal assessment of educational effectiveness. Future development includes incorporation of dynamic perfusion systems to simulate vascular flow and bleeding scenarios, further expanding training opportunities in vascular risk management. In the era of precision oncology, deliberate training in precision surgery—and in coordinated surgical cognition—may be equally essential to sustaining and advancing outcomes for children with cancer.

## Figures and Tables

**Figure 1:**
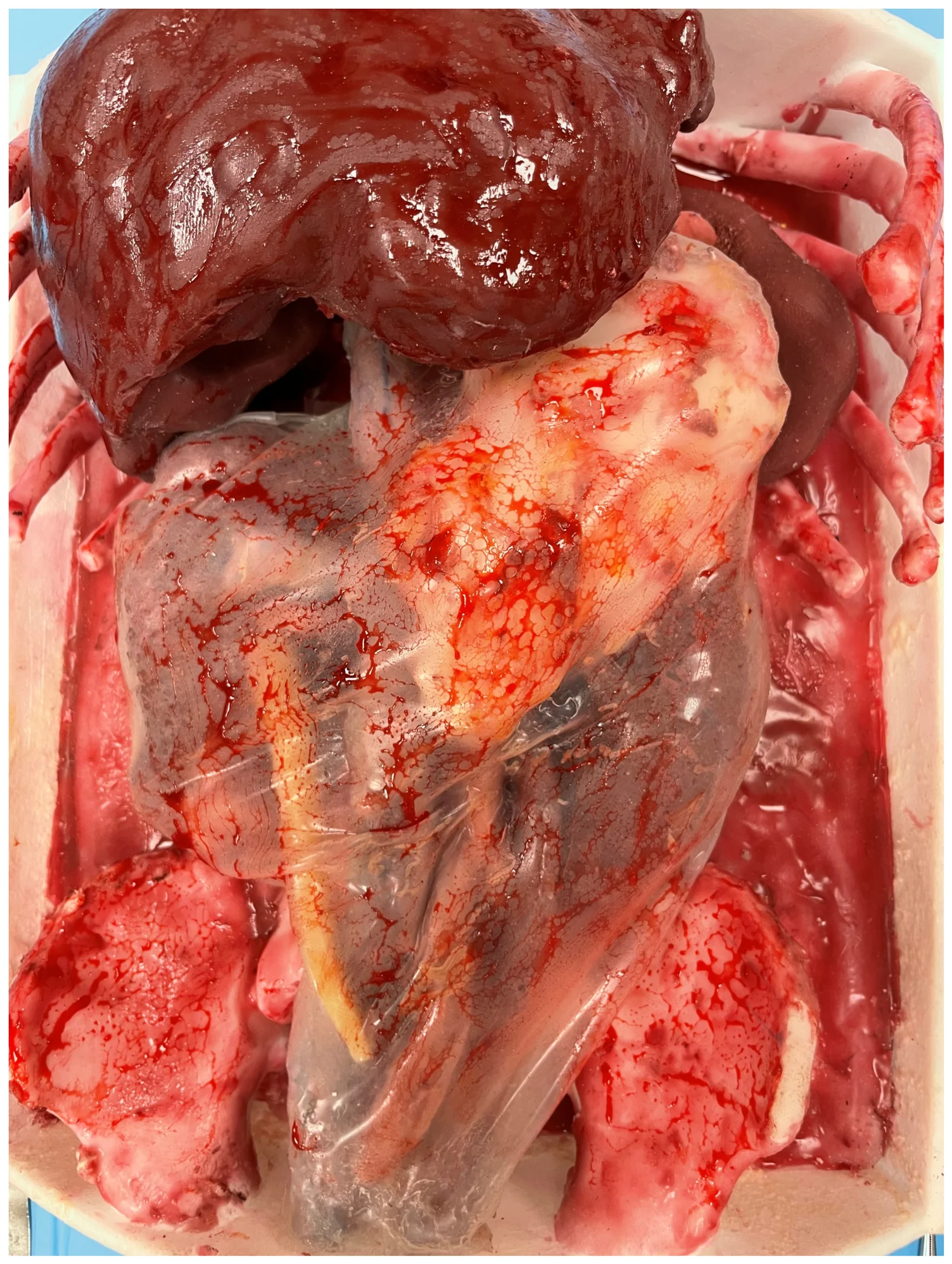
General view of the assembled hybrid simulator inside the housing box, showing the liver, vascular tree, kidneys, and abdominal viscera in their anatomical positions, covered by a thin layer representing the retroperitoneal sheet.

**Figure 2:**
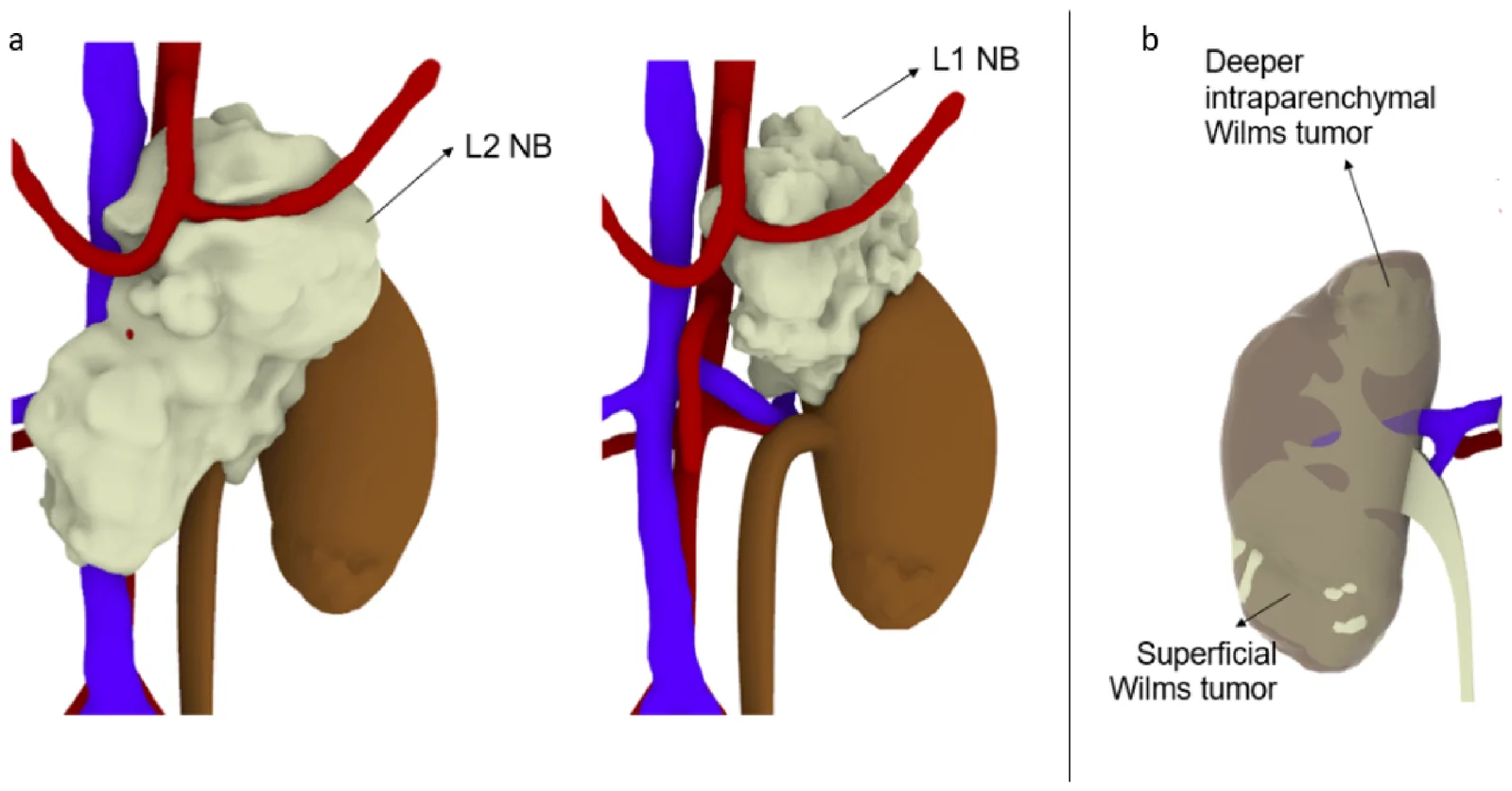
(**a**) L2 and L1 Neuroblastoma. L1 represents a localized tumor without image-defined risk factors, whereas L2 represents a locoregional tumor with image-defined risk factors. (**b**) Deep intraparenchymal tumor focus of the Wilms tumor model, exposed via opening of the renal calyx, located at the superior renal pole in direct contact with the collecting system.

**Figure 3:**
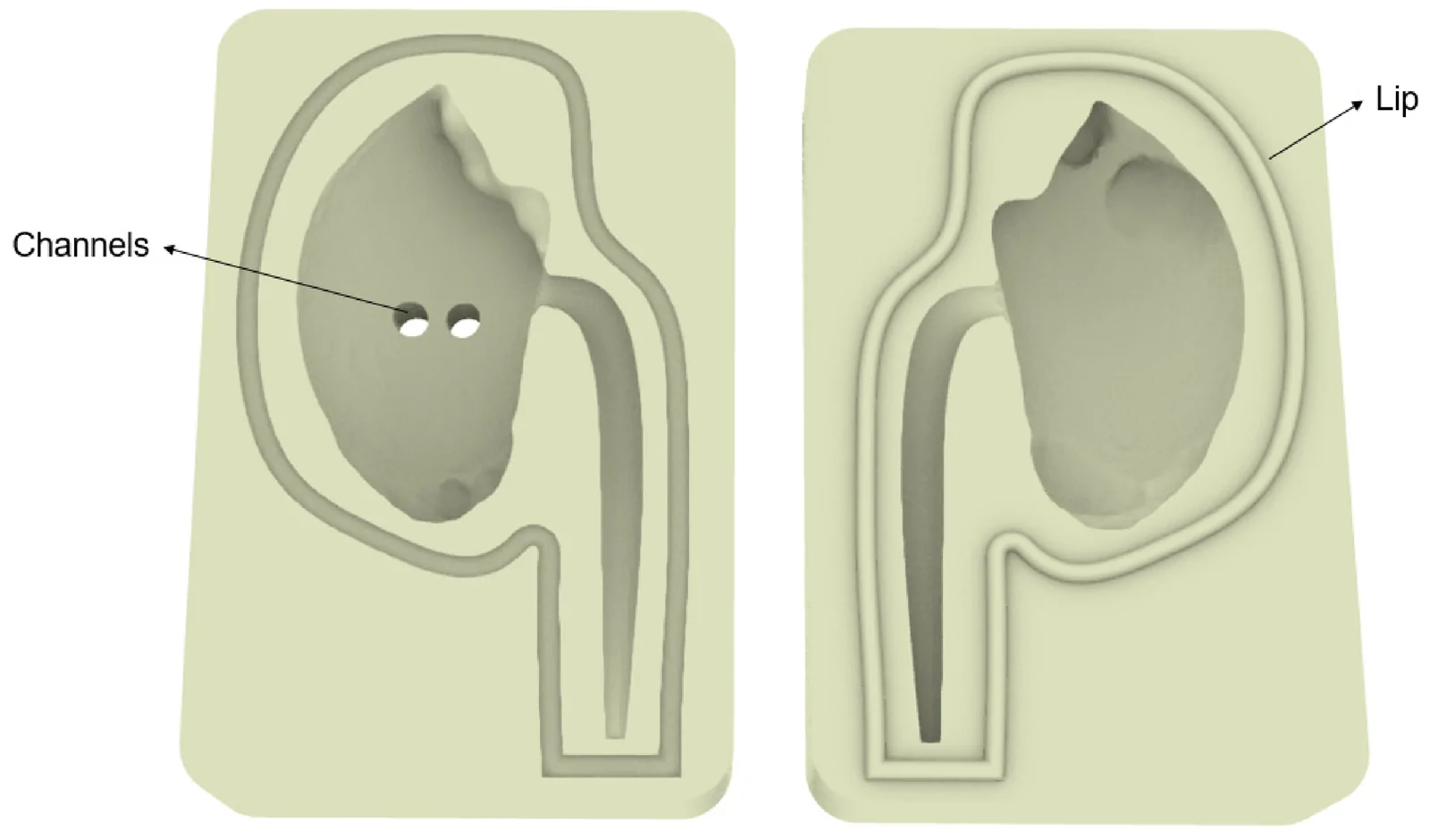
CAD design of the negative-cavity silicone casting mold for the healthy kidney, illustrating the inlet/outlet channel pair (**left**) and the precision-fit alignment lip (**right**) used across all parenchymal organ molds.

**Figure 4:**
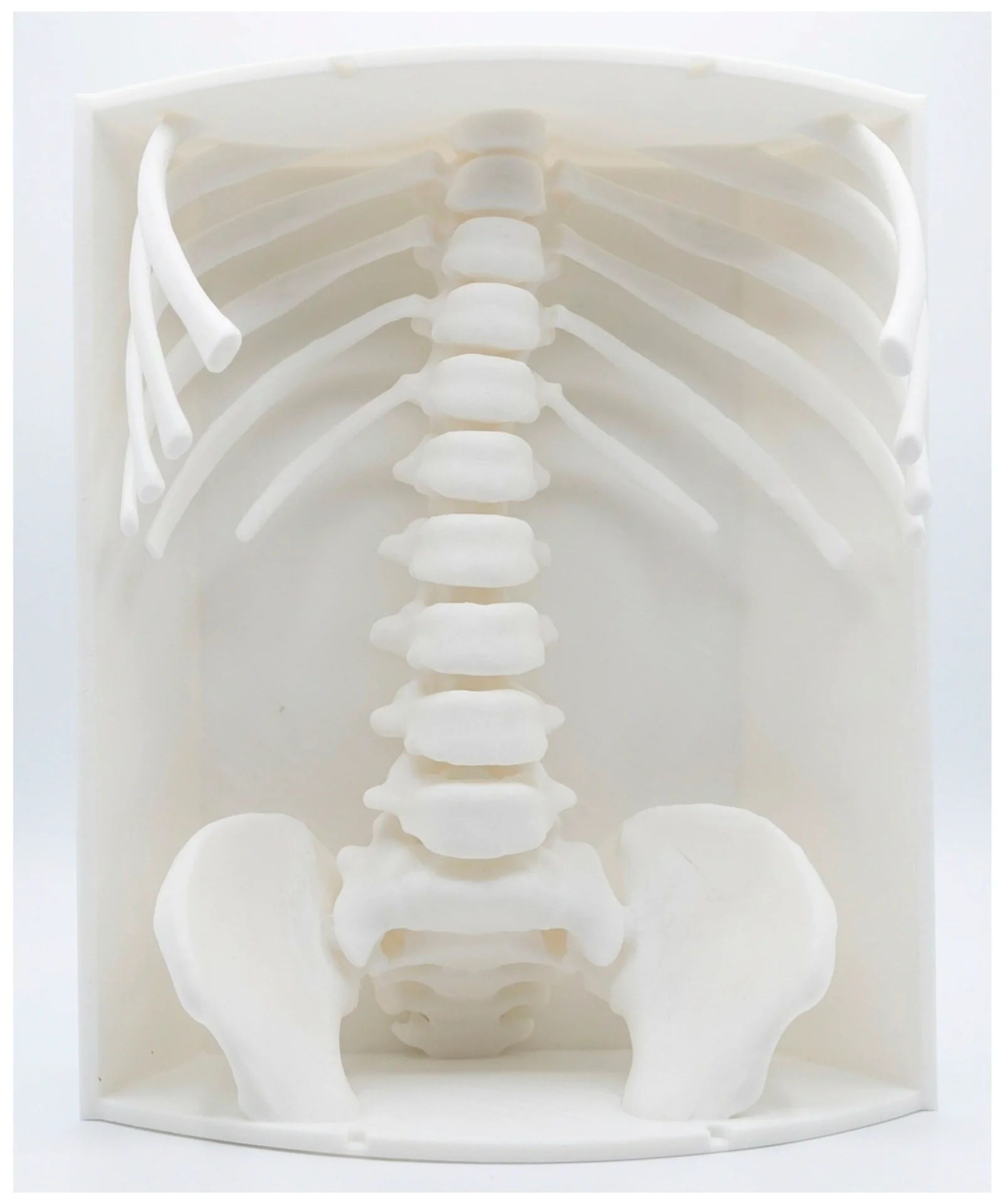
SLS-printed PA12 simulator box with integrated thoracic wall and vertebral column, prior to silicone casting of the dorsal musculature and organ assembly.

**Figure 5:**
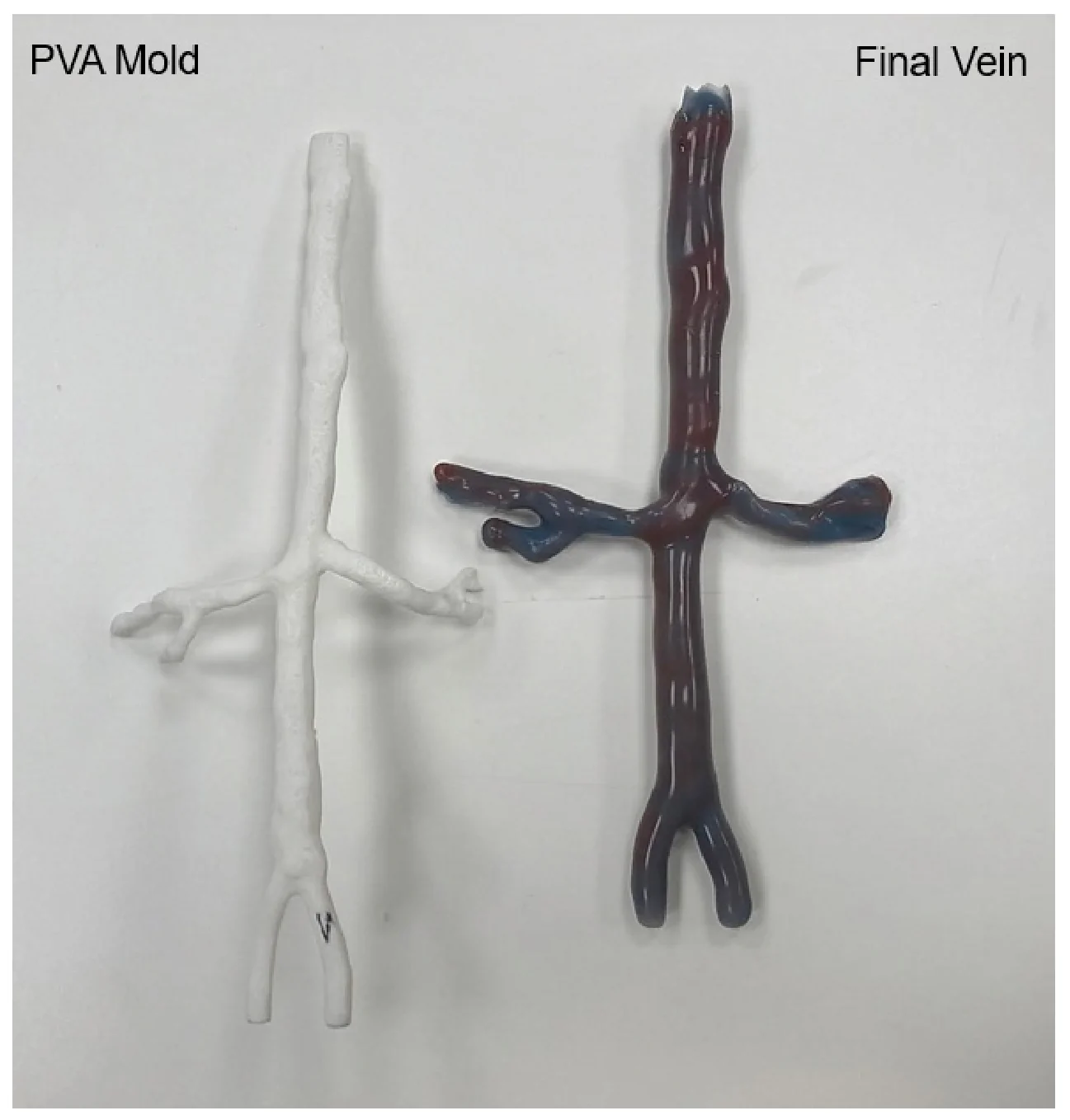
Fabrication of hollow vascular structures over a water-soluble PVA lumen mold: silicone-coated vessels prior to PVA dissolution (white core visible at the proximal ends) and the final patent, compliant vascular tree reproducing the aorta, inferior vena cava, and their branches.

**Figure 6:**
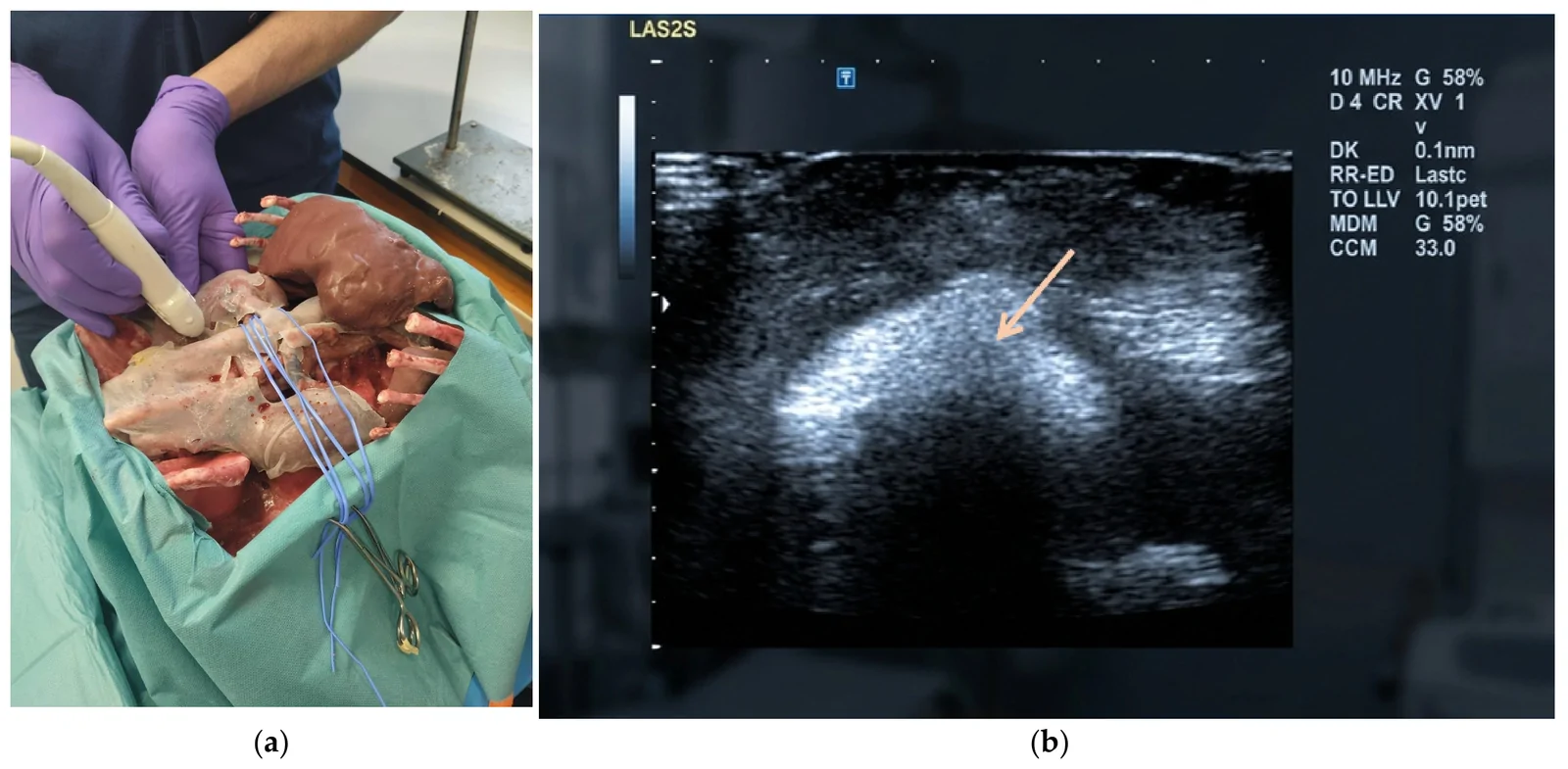
(**a**) Intraoperative ultrasound localization of the corn flour-loaded Wilms tumor focus within the nephron-sparing surgery simulator, reproducing the sonographic detection step required for the deep intraparenchymal lesion. (**b**) An ultrasound view of the Wilms tumor. The arrow demonstrates the location of the lesion.

**Figure 7:**
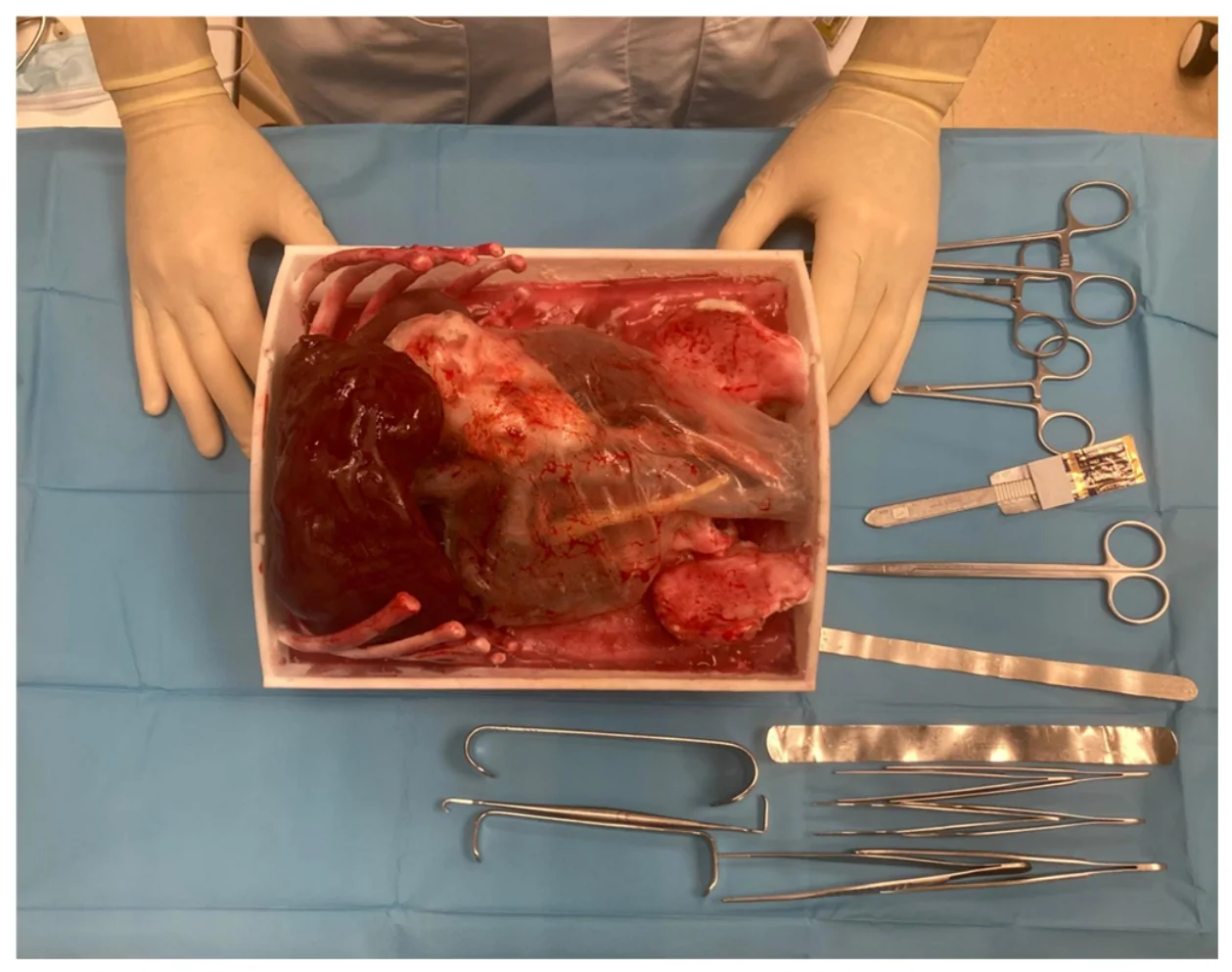
Hybrid simulator and standard open surgical instrument set prepared for a validation workshop session with pediatric surgical oncologists.

**Figure 8:**
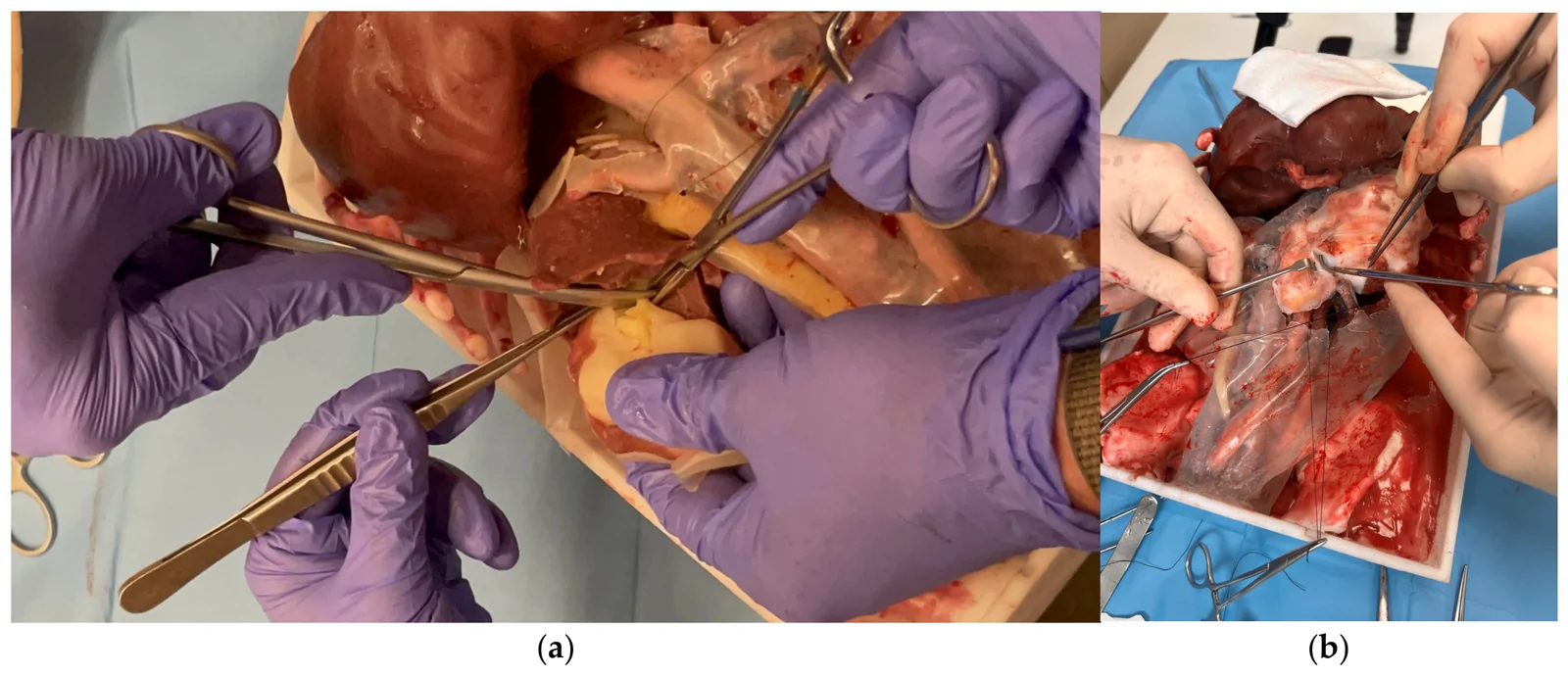
A pediatric surgical oncologist performing simulated dissection during nephron-sparing surgery rehearsal on the simulator (**a**) and renal vessel encasement in neuroblastoma (**b**), demonstrating tissue handling and instrument use during validation workshops.

**Table 1:** Shore hardness of abdominal organs from the literature and corresponding platinum-cure silicone formulations selected for simulator fabrication. Organ data are from Tejo Otero et al. [19] (ovine organs) and Maclean et al. [21] (bovine aorta). Dashes indicate structures for which published hardness data were unavailable; silicone selection was guided by clinical tactile consensus.

Organ	Shore 00 (Literature)	Shore 000 (Literature)	Silicone Selected	Shore (Silicone)
Kidney	32	58	Ecoflex 00-20	Shore 00-20
Liver	18	47	Ecoflex 00-20	Shore 00-20
Spleen	7	40	Ecoflex 00-20	Shore 00-20
Tumor lesion	—	—	Ecoflex 00-30	Shore 00-30
Urinary tract	—	—	Ecoflex 00-10	Shore 00-10
Blood vessels (aorta, IVC, renal vessels)	46	N/A	Dragon Skin 10 Fast	Shore A 10
Intervertebral discs/dorsal muscles	—	—	Soma Foama 25	Ultrasoft foam

**Table 2:** Anatomical components of the hybrid simulator: silicone formulation, mold strategy, mold fabrication material, and box/frame manufacturing method.

Anatomical Structure	Silicone Formulation	Mold Strategy	Mold Material	Box/ Frame
Blood vessels (aorta, IVC, renal arteries)	Dragon Skin 10 Fast (Shore A 10)	Positive lumen mold	PVA—FFF	—
Kidneys	Ecoflex 00-20 (Shore 00-20)	Negative mold	PLA—FFF	—
Tumor (NB/Wilms)	Ecoflex 00-30 (Shore 00-30)	Negative mold	PLA—FFF	—
Liver	Ecoflex 00-20 (Shore 00-20)	Negative mold	PLA—FFF	—
Spleen	Ecoflex 00-20 (Shore 00-20)	Negative mold	PLA—FFF	—
Urinary tract/ collecting system	Ecoflex 00-10 (Shore 00-10)	Negative mold	PLA—FFF	—
Abdominal viscera + mesenteric sheet	Dragon Skin 10 (Shore A 10) + silicone sheet	Negative mold + sheet cast	PLA—FFF	—
Intervertebral discs + dorsal muscle layer	Soma Foama 25 (ultrasoft foam)	Negative mold/direct cast	PLA—FFF	—
Retroperitoneal layer	Ecoflex 00-10 (Shore 00-10)	No mold is required	—	
Simulator box + thoracic wall + pelvic rim + lid	PA12 polyamide (rigid)	—	—	SLS (PA12)

**Table 3:** Mean Likert scores (±SD) across validation domains, pooled across the three simulator models (*n* = 27 model evaluations from 15 participants; see Table 4 for scores disaggregated by model).

Domain	Mean Score (1–5)	SD
Anatomical accuracy	4.14	0.53
Dissection realism	4.29	0.45
Tactile fidelity	4.37	0.56

Note: Because Likert data are ordinal and the sample is small, domain scores are additionally reported as median (interquartile range, IQR): anatomical accuracy, median 4.00 (IQR 3.67–4.67); dissection realism, median 4.33 (IQR 4.00–4.50); tactile fidelity, median 4.00 (IQR 4.00–5.00). Anatomical accuracy and dissection realism are composite scores averaging multiple related questionnaire items per participant (item composition detailed in Section 2.9 and Supplementary Material S1); tactile fidelity remains a single-item score, as the questionnaire included only one item addressing tactile properties.

**Table 4:** Mean Likert scores (± SD) disaggregated by simulator model.

Domain	Wilms Tumor NSS (n = 10)	Neuroblastoma L1 (n = 12)	Neuroblastoma L2 (n = 5)
Anatomical accuracy	4.23 ± 0.57	4.17 ± 0.44	3.87 ± 0.65
Dissection realism	4.38 ± 0.38	4.29 ± 0.43	4.09 ± 0.62
Tactile fidelity	4.40 ± 0.70	4.42 ± 0.51	4.20 ± 0.45

Note: Median (IQR) by model—Anatomical accuracy: Wilms NSS 4.17 (3.67–4.67), neuroblastoma L1 4.00 (3.92–4.42), neuroblastoma L2 3.67 (3.67–3.67). Dissection realism: Wilms NSS 4.27 (4.05–4.57), neuroblastoma L1 4.40 (4.00–4.50), neuroblastoma L2 4.20 (3.50–4.25). Tactile fidelity (single-item): Wilms NSS 4.50 (4.00–5.00), neuroblastoma L1 4.00 (4.00–5.00), neuroblastoma L2 4.00 (4.00–4.00). Anatomical accuracy and dissection realism are composite scores averaging multiple related questionnaire items per participant; see Section 2.9 and Supplementary Material S1 for item composition.

**Table 5:** Participant-level coverage across the three simulator models (*n* = 15 unique participants).

Coverage Pattern	*n* Participants
Evaluated all three models (Wilms NSS, neuroblastoma L1, neuroblastoma L2)	1
Evaluated Wilms tumor NSS and neuroblastoma L1 only	9
Evaluated neuroblastoma L1 only	1
Evaluated neuroblastoma L1 and neuroblastoma L2 only	2
Evaluated neuroblastoma L2 only	2

## Data Availability

The original contributions presented in this study are included in the article/Supplementary Material. Further inquiries can be directed to the corresponding author.

## Supplementary Materials

The following supporting information can be downloaded at: https://www.mdpi.com/article/10.3390/cancers18193135/s1, Supplementary Material S1. Validation Questionnaire.
