# Supplementary material for: Development and Initial Validation of High-Fidelity 3D Hybrid Simulators for Training in Complex Pediatric Oncologic Surgery
Source: Cancers (Basel). 2026 Sep 28;18(19):3135. doi: 10.3390/cancers18193135 (PMC13664311; doi:10.3390/cancers18193135)
Supplement: Supplementary file 1 [file cancers-18-03135-s001.zip › cancers-4525585-Supplementary Material S1. Validation Questionnaire.pdf]

SJD

Sant Joan de Déu
Barcelona · Hospital

Nephron-Sparing Surgery Simulator in Wilms Tumor

Centre ____

Name ____

This comprehensive version draws from validation surveys used in similar surgical simulations and provides targeted questions for evaluating the simulator's realism, educational value, ultrasound integration, and overall effectiveness.

General questions (Yes/No)

Have you trained surgery on a simulator? Yes/No

Have you specifically trained NSS in any setting other than a real clinical context? Yes/No

Have you specifically trained NB in any setting other than a real clinical context? Yes/No

Are you currently involved in surgical education? Yes/No

1. Anatomical accuracy: 1 Poor – 5 Excellent

- Realism of size and tissue characteristics
- Realism of kidney anatomy
- Realism of vascular structures
- Realism of tactile feedback during surgery
- Overall: 3D representation of kidney anatomy and pathology

2. Realism of the procedural steps: 1 Poor – 5 Excellent

- Kidney dissection
- Manipulation of the tissues.
- Suturing
- Depth perception
- Instrument application

3. Simulator Experience Evaluation: 1 (Strongly Disagree) to 5 (Strongly Agree)

- The simulator allowed for the practice of precise tumor resection while sparing the nephron.
- The complexity level of the simulated cases was appropriate for training.
- The simulator included appropriate learning objectives for nephron-sparing surgery.
- The simulator allows for practicing all the essential steps of the surgery

4. Educational Value and Training Effectiveness: 1 (Not at All Effective) to 5 (Extremely Effective)

- The simulator enhanced my understanding of surgical strategies in nephron-sparing procedures.
- It improved my decision-making skills for handling intraoperative challenges.
- The simulator could be useful in the planning of the more appropriate tumour resection technique (enucleation vs enucleoresection vs partial nephrectomy)
- It supported the development of both basic and advanced surgical techniques.

5. Usefulness of Ultrasound (US) and other images Integration: 1 (Not Useful at All) to 5 (Extremely Useful)

- US guidance was intuitive and well-integrated into the simulation.
- The ability to visualize tumors and surrounding structures using US was useful for the tumor localization.
- US-assisted navigation during tumor resection was beneficial for preserving healthy tissue.
- The simulator provided sufficient training on interpreting US findings during surgery.

6. Comparison with Other Training Methods: 1 (Much Worse) to 5 (Much Better)

- The simulator was more effective than traditional cadaver or animal models for practicing nephron-sparing techniques.
- The use of 3D and holographic visualization added a unique and beneficial dimension to training.

7. Overall Usability and Feedback: 1 (Much Worse) to 5 (Much Better)

- The simulator was user-friendly and easy to use.
- The layout and design supported an effective learning experience.
- Overall satisfaction with the simulator.

8. Open-Ended Feedback:

- What aspects of the simulator did you find most valuable?

- What challenges did you face when using the simulator?

- What improvements would you suggest for future versions?

Neuroblastoma L1 Surgery Simulator

Below is a questionnaire for a neuroblastoma L1 surgery simulator, drawing on insights from previous studies and feedback strategies to ensure comprehensive coverage of key aspects. This version is designed to provide a thorough evaluation of the simulator's realism, educational value, imaging capabilities, and its effectiveness compared to other training methods.

1. Anatomical accuracy: 1 Poor – 5 Excellent

- Realism of size and tissue characteristics
- Realism of vascular structures
- Realism of tactile feedback during surgery
- Overall: The simulator provided an accurate 3D representation of neuroblastoma anatomy and surrounding tissues

2. Realism of the procedural steps: 1 Poor – 5 Excellent

- Tumour dissection
- Manipulation of the tissues.
- Suturing
- Depth perception
- Instrument application.

3. Simulator Experience Evaluation: 1 (Strongly Disagree) to 5 (Strongly Agree)

- The simulator captured the complexity of neuroblastoma resection effectively.
- The simulation included critical steps relevant to neuroblastoma surgery.

4. Educational Value and Training Effectiveness: 1 (Not at All Effective) to 5 (Extremely Effective)

- The simulator enhanced my knowledge of neuroblastoma surgical strategies.
- It improved my decision-making skills in complex surgical scenarios.
- It supported learning in both basic and advanced surgical techniques.

5. Comparison with Other Training Methods: 1 (Much Worse) to 5 (Much Better)

- Compared to traditional training methods (e.g., cadaveric models, live demonstrations), how effective was this simulator?
- The simulator's educational impact compared to real-time surgical observation.

6. Overall Usability and Feedback: 1 (Much Worse) to 5 (Much Better)

- The simulator was user-friendly and easy to navigate.
- The layout, design, and step-by-step guidance were conducive to an effective learning experience.
- Overall satisfaction with the simulator (Rate from 1 to 5).
-

7. Open-Ended Feedback:

- What aspects of the neuroblastoma simulator did you find most beneficial for your training?

- What were the main challenges or limitations you encountered while using the simulator?

- Please suggest improvements for future versions of the neuroblastoma simulator.

SJD

Sant Joan de Déu
Barcelona · Hospital

Neuroblastoma L2 Surgery Simulator

Below is a questionnaire for a neuroblastoma L2 surgery simulator, drawing on insights from previous studies and feedback strategies to ensure comprehensive coverage of key aspects. This version is designed to provide a thorough evaluation of the simulator's realism, educational value, imaging capabilities, and its effectiveness compared to other training methods.

1. Anatomical accuracy: 1 Poor – 5 Excellent

- Realism of size and tissue characteristics
- Realism of vascular structures
- Realism of tactile feedback during surgery
- Overall: The simulator provided an accurate 3D representation of neuroblastoma anatomy and surrounding tissues

2. Realism of the procedural steps: 1 Poor – 5 Excellent

- Tumour dissection
- Manipulation of the tissues.
- Suturing
- Depth perception
- Instrument application.

3. Simulator Experience Evaluation: 1 (Strongly Disagree) to 5 (Strongly Agree)

- The simulator captured the complexity of neuroblastoma resection effectively.
- The simulation included critical steps relevant to neuroblastoma surgery.

4. Educational Value and Training Effectiveness: 1 (Not at All Effective) to 5 (Extremely Effective)

- The simulator enhanced my knowledge of neuroblastoma surgical strategies.
- It improved my decision-making skills in complex surgical scenarios.
- It supported learning in both basic and advanced surgical techniques.

5. Comparison with Other Training Methods: 1 (Much Worse) to 5 (Much Better)

- Compared to traditional training methods (e.g., cadaveric models, live demonstrations), how effective was this simulator?
- The simulator's educational impact compared to real-time surgical observation.

6. Overall Usability and Feedback: 1 (Much Worse) to 5 (Much Better)

- The simulator was user-friendly and easy to navigate.
- The layout, design, and step-by-step guidance were conducive to an effective learning experience.
- Overall satisfaction with the simulator (Rate from 1 to 5).
-

7. Open-Ended Feedback:

- What aspects of the neuroblastoma simulator did you find most beneficial for your training?

- What were the main challenges or limitations you encountered while using the simulator?

- Please suggest improvements for future versions of the neuroblastoma simulator.
